# Toward crustacean without chemicals: a descriptive analysis of consumer response using price comparisons

**DOI:** 10.3402/fnr.v60.30955

**Published:** 2016-10-28

**Authors:** Charles Odilichukwu R. Okpala, Gioacchino Bono, Vito Pipitone, Sergio Vitale, Leonardo Cannizzaro

**Affiliations:** Istituto per l’ Ambiente Marino Costiero, Consiglio Nazionale delle Ricerche, Mazara del Vallo, Italy

**Keywords:** consumers, crustacean product, chemical-free packaging, chemical additives, price, stakeholders

## Abstract

**Background:**

To date, there seems to be limited-to-zero emphasis about how consumers perceive crustacean products subject to either chemical and or non-chemical preservative treatments. In addition, studies that investigated price comparisons of crustacean products subject to either chemical or chemical-free preservative methods seem unreported.

**Objective:**

This study focused on providing some foundational knowledge about how consumers perceive traditionally harvested crustaceans that are either chemical-treated and or free of chemicals, incorporating price comparisons using a descriptive approach.

**Design:**

The study design employed a questionnaire approach *via* interview using a computer-assisted telephone system and sampled 1,540 participants across five key locations in Italy. To actualize consumer sensitivity, ‘price’ was the focus given its crucial role as a consumption barrier. Prior to this, variables such as demographic characteristics of participants, frequency of purchasing, quality attributes/factors that limit the consumption of crustaceans were equally considered.

**Results:**

By price comparisons, consumers are likely to favor chemical-free (modified atmosphere packaging) crustacean products amid a price increase of up to 15%. But, a further price increase such as by 25% could markedly damage consumers’ feelings, which might lead to a considerable number opting out in favor of either chemical-treated or other seafood products. Comparing locations, the studied variables showed no statistical differences (*p*>0.05). On the contrary, the response weightings fluctuated across the studied categories. Both response weightings and coefficient of variation helped reveal more about how responses deviated per variable categories.

**Conclusions:**

This study has revealed some foundational knowledge about how consumers perceive traditionally harvested crustaceans that were either chemical-treated or subject to chemical-free preservative up to price sensitivity using Italy as a reference case, which is applicable to other parts of the globe.

Together with supplementary activities such as processing, packaging, and distribution, the fishery sector provides livelihood and income to hundreds of millions of people and accounts for about 10–12% of the world's population. Previous reports show the estimated economic value of global fishery business as of 2012 at US$232 billion. Alongside, crustaceans are among the well-known seafood products of economic and global importance that serve as a source of long-chain polyunsaturated fatty acids (LCPUFAs, or omega-3), including eicosapentaenoic acid and docosahexaenoic acid. Hence, seafood products are well associated with immense health benefits, for example, decreasing the risk of cardiovascular diseases (1–10). On the contrary, there are two major influences that associate with the consumption of fishery products, that is, drivers and barriers. Drivers entail eating habits, perceived health benefits, and sensory likings. Barriers entail high price perception, lack of available preferred products, and either the knowledge or suitability of seafood selection and preparation, as well as sensory disliking (10). Considering this kind of market (fishery products) however, a better understanding of consumer purchasing behavior is very essential if more effective marketing and policy strategies are to be developed. On the other hand, modified atmosphere packaging (MAP) is among chemical-free food process technologies increasingly adapted on commercial fronts as a promising preservation technique, bringing about advances in distribution, storage, and marketability of (raw and processed) fishery products to meet up with consumer demand (2, 11–14). MAP simply involves the modification of gas mixtures within the packaging headspace, mainly, decreasing oxygen contents, while increasing carbon dioxide (CO_2_) contents and/or nitrogen (N_2_) aimed at prolonging shelf life particularly of perishable food products (15). Many seafood products subject to MAP combined treatments have been reported such as sardines (11), maatjes herring (16), lingcod fillets (17), red mullet (13), deep water rose shrimp (13), tiger prawn (18), Pacific white shrimp (14, 19), common dolphin fish (13), and giant red shrimp (20). The use of MAP also aims to supplement the use of chemical additives such as sulphite agents largely applied to crustacean products given its potentials to inhibit postmortem black spot. The use of (such) chemicals that is on the rise has brought about increasing global (public) attention given the health risks associated with its deposits on crustacean flesh. If care is not taken, it can reach above the acceptable residual limits as prescribed by law (20, 21).

To date, there seems to be limited-to-zero emphasis about how consumers perceive crustacean products subject to either chemical and or non-chemical preservative treatments. In addition, studies that investigated price comparisons of crustacean products subject to either chemical or chemical-free preservative methods seem unreported. Such price comparisons would not be limited to the different preservative methods applied to crustacean products, but can extend to how consumers perceive these different preservative methods. Although the use of chemical preservatives, for example, sulphite agents, as well as chemical-free preservative methods, for example, MAP, applied to crustacean products remain increasingly investigated, how consumers perceive these preservative methods seems largely neglected. To achieve this would require the use of knowledge, experience, and attitudes of consumers/individuals, as described by Zanin et al. (22). Largely, consumers can be of two types, that is, usual and unusual. Usual refers to those that consume products regularly over a given time period. Unusual refers to those classed as occasional consumers with more inconsistent or varying consumption patterns. Equipping consumers with the prerequisite knowledge as well as the means can help them make informed food choices. Following this, some factors such as information about the product, attitudes, and beliefs, as well as past experiences can directly or indirectly affect their food-related decisions (23, 24). Also, the use of different preservative methods may well result in differences in product price, quality, and quality, which in this context makes the concept of price and product quality very pertinent. Because treating crustacean products with a chemical-free method to improve shelf life would predictably affect/increase the eventual selling price to the detriment of consumer pay, a number of relevant questions very pertinent for the end user are reckonable, such as: 1) In what way would consumers of crustacean products comparatively perceive either of these chemical and chemical-free preservation methods (such as MAP)? 2) In what way do the consumers of crustacean products actually respond to the price effect of these preservation methods? 3) Could this be directly or indirectly related to attitude, knowledge, and experience of consumers with regard to the preservation methods vis-à-vis crustacean products? For the reason that both attitude and knowledge are considered to correlate positively (22, 25), to understand how and to what degree consumers would respond to the above-highlighted questions would be worthwhile. Assembling all the above-highlighted discourse would make a strong case for why chemical-treated and MAP vis-à-vis consumer types need additional studies. Consistent with the global quest of going ‘green’, as seafood industries, stakeholders, and responsible policymakers move from chemical onto any of the existent food technologies such as MAP, they would require thorough foundational knowledge about consumption patterns of consumers toward crustacean products prior to understanding its perception not only for processed products but also the associated/corresponding technology. This would help better their understanding about consumers’ appreciation of the emergent processed products. In line with a move toward crustaceans without chemicals, the authors believe that a study that lays foundational knowledge about consumer sensitivity of chemical-treated and non-chemical-treated (MAP) crustacean products will help supplement existing information.

In this study therefore, in order to establish foundational knowledge about consumers of crustacean products, and prior to the conduct of any form of consumer sensitivity studies into crustacean product(s), we started by giving full attention to consumption types and frequency, quality attributed to or associated with, as well as factors promoting/limiting consumption of crustacean products. Because relevant information about sensitivity of consumers to fishery products subject to chemical and chemical-free preservation (such as MAP) methods appears unreported, we will also consider consumer sensitivity by price comparisons between chemical and non-chemical preserved (MAP) crustacean products.

## Methods

### Consumers and sampled population

The conducted study targeted adult (aged 18 years old and above) (usual and unusual) consumers of crustacean products. Prior to participation, informed consent was verbally sought from participants by interviewers. All participants were made aware that information would be coded and all personal information would be treated with a high level of confidentiality. In addition, the interviewees (consumers) of this work had neither direct nor indirect contact with the authors during the interview process.

Five locations deemed representative of Italy were sampled namely Palermo, Naples, Rome, Milan, and Turin. In total, 1,540 consumers participated. Majorly, Palermo and Naples represent southern, Rome represents central, and Turin and Milan represent northern parts of Italy. Using the estimates of September 2014 made available by National Institute of Statistics of Italy (ISTAT) that is widely accessible online (www.istat.it/en), the selected locations cumulate to over 14 million of Italy population.

### Research instrument

Based on expertise and experience of the authors, the questionnaire that served as the instrument of this study was developed and structured in such a way to cater to the usual and unusual consumer types. Apart from the preliminary section that constituted assessment of demographic characteristics in terms of gender, age, household composition, and educational level of interviewees and their partners, the main questions (which did not change much after the validation process) presented to participants through interview are detailed below.

1. *Major section 1*: To the consumers of crustacean products of this study, the following major questions were presented: 1) Do you consume crustacean products? 2) If yes, how often do you consume crustacean products? 3) What attributes do you use to qualify crustacean products at the point of purchase? 4) If you and your family usually consume crustaceans, may we know the reasons that guide your consumption? 5) At which place do you usually purchase crustacean products? 6) What quantity of crustacean products do you purchase at any given time? 7) Given that you purchase crustacean products, do you either think it contains chemicals or it was/has been preserved with chemicals? 8) If you believe it contains chemicals, do you think it would pose any danger at all? 9) If you believe it contains chemicals, do you think it would worsen the taste of the product? 10) If you believe it contains chemicals, do you think it would worsen the odor of the product?
2. *Major section 2*: Specific to occasional consumers of crustacean products of this study, the following major questions were presented: 1) Can you reveal the reasons that limit your consumption of crustacean products? 2) If you were hinted/informed that the crustacean product was preserved with chemicals, would you see the product as unhealthy?
3. *Major section 3*: To elucidate consumer sensitivity to price as a start, two scenes of price estimates between the chemical and chemical-free preserved crustacean products were presented. ‘Price’ is selected as a start because it is a crucial consumption barrier that if it attains an unexpected peak may rapidly receive unfavorable consumer response. The authors feel it is necessary to make readers pay good attention to ‘price’ as they reflect on how respective consumers responded to the chemical and chemical-free (MAP) crustacean products.

The two scenes are hereby detailed as follows:

1. *Scene 1*: Consumers are presented with the question of whether they would purchase crustaceans preserved with chemicals with estimated price of €15/kg. At the same time, consumers are presented an alternative that is chemical-free that estimated price increased by 15%.
2. *Scene 2*: At this scene, a similar question as scene 1 is presented but with increased price of €25/kg for crustaceans preserved with chemicals. At the same time, the alternative of chemical-free is presented with estimated price increased by 25%.

In the context of the above scenes, usual consumers were consequently asked: 1) Because the chemical-free preserved crustacean products are priced higher, what adjustments are you willing to make to overcome the challenge? On the other hand, the unusual consumers were given the option of: 2) Would you go for another seafood product? These scenes have been aimed to elucidate the degree of sensitivity of consumers to the different price increases that could be anticipated in a real-time scenario. This allowed for consumers to express themselves and respond to concerns of price.

### Validation of research instrument

The specialized companies on demographic studies namely: SWG S.p.A. (Trieste, Italy) and Demopolis S.r.l. (Palermo, Italy) collaboratively validated the study instrument. The outcome helped to modify the questions prior to the actual interview process. Responses from the validation exercise did not form any part of the main study. Both abovementioned companies specialize in studies on demographic dynamics and behavior, opinion, and sector studies.

### Research process and data collection

The prerequisite conditions set for respondents’ participation included: 1) to be at least 18 years old; and 2) to be part of a household. Trained professional interviewers of SWG S.p.A. (Trieste, Italy) constituted the interview panel and also implemented the actual interview process of this study. The interview panel underwent specific training based on the study variables/variable categories. The interview process involved the use of a computer-assisted telephone interview system that spanned over an 8-week period. Computer-assisted telephone approach is not new in agro/food-based population studies as it is believed to show satisfactory outcomes. Similar to a published report and using random digit telephone number dials, the interview panel reached out to as many participants as possible. At different times of the day, calls were made and if there was no answer, it was subsequently followed by at least three other attempts. If a call got answered but the eligible person was not available, the interviewer would request a specific time for repeat call and make a few subsequent attempts (26). The professional experts of SWG S.p.A. (Trieste, Italy) assembled the resultant data and weighted size of respondents. Importantly, data has been coded for anonymity that excluded all consumers’ personal information, which allowed for only the consumers’ responses to questions relevant to this study.

### Statistical analysis

All resultant data were subject to normality test. The output of normality test confirmed the data were non-parametric. As a consequence, Kruskal–Wallis (K-W) analysis of rank test was applied to establish whether any statistical differences existed in outcomes/responses per variable/variable category. Where data of variable categories (within the studied variables) required comparisons, Student's *t* and Fisher's exact probability tests were applied. According to the research questions, the responses were categorized and depicted as variables/variable categories consistent to all locations, tabulated, and represented in percentages (Tables 1–5). Spearman's correlation test was applied in order to find out whether any associations existed between the measured variables. Probability level of *p*<0.05 was considered statistically significant. Minitab Express software v.1.2.0. (Minitab Ltd., Coventry CV3 2TE, UK) was used to do the statistical analysis.

**Table 1:** Demographic characteristics of respondents by gender, age, household composition, and educational levels across locations

Variable	Variable category	*n*	Palermo (%)	Naples (%)	Rome (%)	Milan (%)	Turin (%)	Mean (%)	CV	*p*^a^
Gender	Male	438	29.9	23.9	29.4	28.6	29.6	28.28	8.8	>0.05
	Female	1,102	70.1	76.1	70.6	71.4	70.4	71.72	3.5	
Age (years)	18–24	45	2.8	2.8	2.8	2.9	3.6	2.98	11.7	>0.05
	25–34	164	10.6	10.8	13.3	10.0	6.8	10.30	22.6	
	35–44	362	24.4	27.1	18.6	17.7	27.6	23.08	20.3	
	45–54	291	24.0	23.9	20.9	18.3	21.6	21.74	10.9	
	55–64	349	23.6	17.9	22.1	25.4	16.0	21.00	18.7	
	≥65	329	14.6	17.5	22.3	25.7	24.4	20.90	22.5	
Household composition	1 (interviewee)	134	3.9	4.0	8.7	12.3	13.2	8.42	52.4	>0.05
	2	432	21.3	20.3	30.8	36.0	26.8	27.04	24.3	
	3	379	20.5	21.9	24.6	27.4	27.6	24.40	13.1	
	4	447	36.2	36.3	30.6	18.3	26.8	29.64	25.3	
	5	121	14.2	13.9	4.4	5.4	4.8	8.54	59.1	
	>5	27	3.9	3.6	0.9	0.6	0.8	1.96	83.7	
Education level	Elementary	176	11.0	15.5	10.4	9.7	12.0	11.72	19.4	>0.05
	Junior high	361	28.3	22.7	17.9	24.0	28.0	24.18	17.7	
	Middle level	658	40.6	43.4	48.3	39.4	39.2	42.18	9.0	
	Bachelor	325	19.3	17.9	22.0	24.9	19.2	20.66	13.6	
	Post-bachelor	20	0.8	0.4	1.4	2.0	1.6	1.24	51.5	
Partner's education level	Elementary	102	5.1	8.4	5.7	6.9	7.6	6.74	20.0	>0.05
	Junior high	291	23.6	25.9	14.7	16.3	18.0	19.70	24.5	
	Middle level	491	34.6	31.5	34.9	29.7	27.2	31.58	10.4	
	Bachelor	279	15.7	15.1	18.6	18.9	21.6	17.98	14.7	
	Post-bachelor	11	0.8	1.2	0.7	0.3	0.8	0.76	42.2	
	Without spouse	366	20.1	17.9	25.3	28.0	24.8	23.22	17.7	

*n*=sum of respondents per variable category.

a Outcome of (K-W) analysis of variance test of variables across locations. CV, coefficient of variation.

### Response weightings and coefficient of variation

Response weighting was developed based on the sum of responses across the studied locations per variable category of this study (Tables 1–5). Attributing the response weighting with either ‘ascending’ or ‘descending’ trends is therefore possible because it represented the cumulative total of the actual responses. Notably, respondents of specific variable categories with higher response weightings would not necessarily depict a higher influence of that variable category over another. This is because at a different variable category there could exist a lesser response weighting but with a higher response outcome at a specific location. Coefficient of variation (CV) was also developed as the ratio between the standard deviation and the mean values. Well established, it depicts the extent of deviations in relation to the mean as shown in Tables 1–5. Both response weightings and CV improve the understanding regarding the degree of deviation of responses per variable categories.

## Results and discussion

### Consumer demographic characteristics

The demographic characteristics of respondents by gender, age, household composition, and educational levels across locations are given in Table 1. Comparing locations, the trend of gender, age, household composition, and educational levels appeared similar (*p*>0.05). Regardless of location, females clearly dominated over males (*p*<0.05). Furthermore, the sampled population of middle aged (35–44 years) and elderly (greater than 65 years) appeared the most varied categories across location. In general, youth participation (≤34 years) appeared much less compared with the adults especially those of four persons per household, which also seemed noticeable across location(s). In addition, household composition of between two and four persons appeared more frequent compared with the others. By response weightings, educational level fluctuated, which is somewhat aligned with the CV values. ‘Middle’ level of education was highest by response weighting and proportion of respondents across locations, before ‘junior high’ and before ‘bachelor’. The trend of educational level somewhat resembled those of partners’ education level variable(s) if ‘without spouse’ variable categories are to be excluded. Notably, ‘without spouse’ had fairly above 20%, which should not be neglected. Also, special attention needs to be paid to the overall educational level of respondents as it appeared relatively reasonable at this study, which may likely strengthen the robustness of responses of major sections given that the majority plausibly understood to large extent the questions posed to them during the interview(s). Such would then make their responses reflect the actual real-time situation. Connecting the respective variables and CV values, the higher degree of variability is shown at the male gender, age groups of between 25 and 34 years, household composition greater than five as well as those of post-bachelor educational level (interviewees & partners).

### Consumer types, consumption frequencies, and qualities ascribed to crustacean product

There are consumers who would like to consume much less of fishery products, which can emanate from family influence to negate consumption levels of fishery products (27–29). Plausibly, considerations of contextual setting, personal (values, beliefs, attitudes, and demographics), and situational factors could affect consumer purchasing behavior toward fishery products, particularly when it concerns the relative choice of quantity and frequency of fishery consumption (10). Consumer types, frequency of consumption, and quality attributed to or associated with crustacean products by locations are given in Table 2. Of usual consumers, locations in Palermo followed by Naples received the higher response. Of unusual consumers, locations in Turin followed by Milan received higher response. The result may well suggest some strong fishery cultural root/tradition along coastal locations in Palermo and Naples, which corroborates the anticipated higher consumption of fishery products compared to non-coastal locations in Turin and Milan. Also shown in Table 2, the frequency of consumption of crustacean product found Palermo dominating ‘twice per month’ before Naples and Rome dominating ‘at least once a month’, and Turin dominating ‘never’ before Milan. Previously reported cross-cultural and single cross-sectional studies have underlined that knowledge, skills, and self-confidence in the selection and preparation of fishery products can positively/significantly impact on its consumption frequency (10, 27, 30, 31).

**Table 2:** Consumer types, frequency of consumption, and qualities attributed to or associated with crustacean products across locations

Variable	Variable category	*n*	Palermo (%)	Naples (%)	Rome (%)	Milan (%)	Turin (%)	Mean (%)	CV	*p*^a^
Type of consumers	Usual	747	61.0	58.6	46.4	41.1	40.4	49.50	19.6	>0.05
	Non-usual	793	39.0	41.4	53.6	58.9	59.6	50.50	19.2	
Frequency of consumption	Everyday	0	0.0	0.0	0.0	0.0	0.0	0.00	0.0	>0.05
	2/3 times per week	45	2.0	5.0	3.0	3.0	2.0	3.00	40.8	
	One time per week	149	15.0	18.0	7.0	6.0	6.0	10.40	54.6	
	Twice per month	235	22.0	20.0	13.0	12.0	12.0	15.80	30.5	
	At least once per month	314	21.0	16.0	24.0	19.0	20.0	20.00	14.6	
	At least once every 2/3 months	257	17.0	15.0	18.0	16.0	16.0	16.40	7.0	
	At most twice per year	187	12.0	13.0	12.0	14.0	10.0	12.20	12.2	
	Not more than once per year	91	4.0	3.0	6.0	9.0	6.0	5.60	41.1	
	Never	259	7.0	10.0	17.0	21.0	28.0	16.60	50.9	
Qualities attributed crustacean	Freshness	399	42.8	42.1	43.2	42.2	43.0	42.66	1.1	>0.05
product	Genuineness	126	12.8	11.5	15.6	13.5	13.7	13.42	11.1	
	Odor	109	11.8	12.0	11.0	11.5	12.1	11.68	3.8	
	Taste	55	5.9	7.7	5.7	5.7	4.3	5.86	20.7	
	Color	68	9.0	9.8	4.7	6.8	6.0	7.26	29.1	
	Additive-free	85	7.5	8.7	12.0	8.3	8.8	9.06	19.0	
	Packaging method	67	8.0	6.6	5.2	7.3	8.8	7.18	19.2	
	Healthiness	24	2.2	1.1	2.6	4.2	2.7	2.56	43.6	
	No idea	3	0.0	0.5	0.0	0.5	0.6	0.32	92.2	

*n*=sum of respondents per variable category.

a Outcome of K-W analysis of variance test of variables across locations. CV, coefficient of variation.

Across the abovementioned locations, respondents indicated ‘freshness’ highly as quality attributed/associated to/with the crustacean product somewhat followed by ‘genuineness’ before ‘odor’ particularly if response weightings are to be first considered and less the responses given by location. Respondents of an Australian survey found ‘freshness’ and ‘quality’ of fishery product difficult to assess because some respondents indicated to buying/purchasing more particularly when their confidence to evaluate the product quality had (been) improved (32). In a somewhat similar position, Juhl and Poulsen (33) reported that the less traditional fishery consumers were not able to determine the freshness of fishery products based on appearance and odor compared to the strongly involved fishery consumers. However, connecting the respective variables and CV values, higher degree of variability can be seen at the usual type of consumers, those that indicated ‘one time a week’ as their frequency of consumption well as those that had ‘no idea’ about the quality attributed to crustacean product.

### Usual consumers: reasons for consumption, places of purchase, purchase quantity, and perception to chemicals

The reasons for consumption, places of purchase, estimated quantity per purchase, and perception to odor/taste of chemical-treated crustacean products of usual consumers across locations are given in Table 3. Comparing locations, there were no statistical differences at reasons family consumed crustacean products, place of purchase of crustacean product, quantity of crustacean products purchased, and whether chemicals added posed danger or affected odor and taste (*p*>0.05). Notably, ‘taste’ variable received the highest response followed by ‘family tradition’ before ‘it's healthy’, which interestingly were showed apparently over ‘nutritional value’ of crustacean products. This may necessarily not infer that the sampled population who responded in this manner was not aware of the nutritional benefits the crustacean products brought to their diets. Maybe, at the point of response of the conducted interview, such respondents did not specifically view the nutritional benefits of crustacean products as the top most priority to emphasize. Nonetheless, with regard to the place of purchase of crustacean products, whilst respondents of Palermo and Naples favored the ‘Fish Shop’, those of Rome, Milan, and Turin favored the ‘Supermarket’. This result may possibly highlight the ethnological roots binding the peoples of Naples and Palermo that seemingly associated fish shop with odor of freshly harvested marine products, which contrasts with those of Milan and Turin not seemingly associated with such ethnological roots to plausibly account for (their) increased patronage for ready-to-cook seafood products (of supermarket). As an emerging/dominant force for fishery production, the ‘Supermarket’ has been highly patronized at Milan and Turin concurring with Santulli and Modica (34) who emphasized its accessibility to make seafood products readily available consistent in quality/size to satisfy consumers’ pressing needs. Furthermore, a greater proportion of respondents at Palermo, Naples, and Turin purchased between 500 and 1 kg, whereas Rome and Milan purchased about/around 500 g. More than half of the respondents nevertheless believe that crustacean products contained no chemicals. When asked whether chemical additives added to crustacean products posed any danger, or worsened the taste or odor, more than half of respondents wholly indicated between ‘little’ and ‘enough’ knowledge, with ‘little’ appearing in most cases. Connecting the respective variables and CV values, there was higher degree of variability at those that had ‘no idea’ about why the family consumed crustacean products, ‘non-specialized shop’ as place of purchase of crustacean products, estimated quantity of purchase of between 2 and 3 kg, those that indicated ‘yes’ that crustacean contained chemicals and as well, indicated ‘very much’ to chemicals added to crustacean products either posed danger or worsened taste. Interestingly, there appears some closeness in the CV of between ‘very much’ and ‘just enough’ about the addition of chemical to worsen the odor of crustacean product(s).

**Table 3:** Reasons for consumption, places of purchase, estimated quantity per purchase, and perception to chemical additives either posing danger or affecting the odor/taste of crustacean products by usual consumers across locations

Variable	Variable category	*n*	Palermo (%)	Naples (%)	Rome (%)	Milan (%)	Turin (%)	Mean (%)	CV	*p*^a^
Reason family consume crustacean product	Taste	325	57.4	44.8	60.0	53.9	21.1	47.44	33.3	>0.05
	Diet/quality	39	1.4	4.5	7.3	6.1	12.6	6.38	64.6	
	Acceptable price	18	4.1	2.2	1.4	0.8	6.3	2.96	75.8	
	Nutritional value	50	8.1	11.2	4.0	4.6	11.6	7.90	45.1	
	Family tradition	121	14.9	21.6	14.0	18.5	26.3	19.06	26.5	
	It is healthy	95	13.5	15.7	12.0	13.0	20.0	14.84	21.5	
	Other	8	0.7	0.0	1.3	2.3	2.1	1.28	75.1	
	No idea	1	0.0	0.0	0.0	0.8	0.0	0.16	236.2	
Place of purchase of crustacean product	Fish shop	303	73.6	67.4	37.3	22.5	11.0	42.36	64.7	>0.05
	Supermarket	228	5.4	18.8	39.3	60.2	56.0	35.94	65.7	
	Street market	126	17.6	11.9	21.3	14.0	33.0	19.56	42.6	
	Non-specialized shop	4	1.4	0.0	0.7	0.7	0	0.56	104.6	
	Other	10	2.0	2.1	1.3	1.5	0	1.38	60.9	
Estimated quantity per purchase	<500 g	91	5.2	9.7	13.9	31.8	9.6	14.04	74.0	>0.05
	≅500 g	212	24.8	29.1	36.1	39.7	34.0	32.74	17.9	
	500 g–1 kg	260	50.3	46.2	34.7	23.8	44.7	39.94	26.8	
	1–2 kg	70	15.7	13.4	12.6	3.2	6.4	10.26	51.0	
	2–3 kg	7	2.0	0.8	0.7	0.0	2.1	1.12	80.7	
	No idea	12	2.0	0.8	2.0	1.5	3.1	1.88	44.7	
Crustacean purchased have chemicals?	Yes	175	32.0	20.9	27.2	29.4	23.9	26.68	16.5	>0.05
	No	403	58.2	68.7	59.4	60.3	65.2	62.36	7.1	
	No idea	75	9.8	10.4	13.4	12.7	10.9	11.44	13.5	
Chemical added to crustacean poses	Very much	91	24.3	14.7	17.3	7.1	5.6	13.8	55.6	>0.05
danger?	Just enough	183	29.1	25.7	36.7	24.1	33.3	29.78	17.6	
	Little	231	31.1	44.0	26.7	50.9	44.4	39.42	25.6	
	Not at all	42	4.7	6.4	5.3	8.9	11.1	7.28	36.7	
	No idea	62	10.8	9.2	14.0	8.9	5.6	9.70	31.5	
Chemical added worsens taste of crustacean product?	Very much	95	27.4	15.0	12.0	6.7	10.0	14.22	56.0	>0.05
	Just enough	152	22.6	22.6	24.0	27.5	22.5	23.84	9.0	
	Little	238	30.8	39.9	32.7	41.7	46.1	38.24	16.7	
	Not at all	96	12.3	15.0	20.0	13.3	13.5	14.82	20.6	
	No idea	57	6.9	7.5	11.3	10.8	7.9	8.88	22.8	
Chemical added worsens odor of	Very much	67	14.7	8.3	12.7	8.3	9.3	10.66	27.1	>0.05
crustacean product?	Just enough	151	34.1	22.6	25.3	15.8	23.3	24.22	27.2	
	Little	215	25.6	40.6	25.3	45.0	41.9	35.68	26.6	
	Not at all	121	17.8	18.8	22.7	22.6	14.0	19.18	19.0	
	No idea	64	7.8	9.8	14.0	8.3	11.5	10.28	24.6	

*n*=sum of respondents per variable category.

a Outcome of K-W analysis of variance test of variables across locations. CV, coefficient of variation.

### Unusual consumers: consumption limitations and perception to chemicals

Price is understood as the most cited barrier of consumption of fishery products. Although some authors agree that cheaper prices would encourage higher consumption of fishery products, others somewhat oppose this given the lack of significant differences in consumption of given fishery products, for example, at expensive meal options. Thus, it is not yet clear whether the perception of high price actually affects consumption of fishery products (10, 27, 32, 35). The reasons limiting consumption and health perception of chemical-treated crustacean products by non-usual consumers across locations are given in Table 4. By response weightings, ‘high price’ largely accounted for the limited consumption of crustacean products given by higher responses by location. This occurred in the following order: ‘Naples > Palermo > Rome > Turin > Milan’. Acceptably, this result appears attributable to the long-existing discrepancies in both income earnings and living standards differentiating both northern (Milan and Turin) and southern (Palermo and Naples) geo-parts of Italy. Although those at Turin equally responded between ‘high price’ and ‘unaccustomed’, those of Milan indicated more for ‘unaccustomed’. Concerning the ‘dislike of crustacean product’, Palermo, Naples, and Turin would rank second at variable category. Additionally, the presence of young children and teenagers up to adolescents in households can be considered among important consumption barriers of fishery products (10). Across locations and based on response weightings, the ‘Reason(s) limiting consumption of crustacean’ had its variable category with the following trend: ‘high price’>‘unaccustomed’>‘dislike of product’>‘difficult to cook’>‘doubts about quality and safety’>‘other’>‘allergy concerns’>‘product odor’>‘No idea’.

**Table 4:** Reasons limiting consumption and health perception of chemical-treated crustacean products by non-usual consumers (occasional) across locations

Variable	Variable category	*n*	Palermo (%)	Naples (%)	Rome (%)	Milan (%)	Turin (%)	Mean (%)	CV	*p*^a^
Reason(s) limiting	High price	193	32.4	38.4	31.1	23.0	29.4	30.86	18.0	>0.05
consumption of	Unaccustomed	183	13.2	16.7	21.5	28.5	29.4	21.86	32.6	
crustacean	Dislike of product	130	16.9	19.6	15.6	16.9	14.7	16.74	11.1	
	Doubts about quality and safety	64	14.0	10.9	8.1	4.6	7.4	9.00	39.8	
	Product odor	21	6.6	2.8	2.2	2.3	0.7	2.92	75.4	
	Allergy concerns	29	5.9	2.8	4.4	2.3	3.7	3.82	37.1	
	Difficult to cook	71	5.1	4.4	10.4	12.3	8.1	8.06	41.9	
	Other	57	5.1	4.4	6.7	10.0	6.6	6.56	32.9	
	No idea	1	0.8	0.0	0.0	0.0	0.0	0.16	223.8	
Crustacean with	Very much	185	38.6	32.7	21.5	14.1	22.1	25.8	37.8	>0.05
chemicals:	Enough	283	35.6	31.7	37.8	37.9	32.2	35.04	8.5	
unhealthy?	Little	168	11.9	20.2	20.6	26.2	22.1	20.2	25.8	
	Not at all	40	4.0	3.8	3.4	9.2	3.4	4.76	52.4	
	No idea	117	9.9	11.5	16.7	12.6	20.1	14.16	29.4	

*n*=sum of respondents per variable category.

a Outcome of K-W analysis of variance test of variables across locations. CV, coefficient of variation.

Although regular consumption of fishery products has been linked to health and nutritional benefits, chemicals and other associated contaminants should not be left out (36). Table 4 also reveals the responses regarding whether respondents deemed chemical-treated crustacean products as ‘unhealthy’ across locations. More than half of respondents seemed conscious about health risks that associate with chemical additives of crustacean products, which might relate with the peak response of 73% at Palermo and by response weightings also, this feat seems reflected by ‘enough’ before ‘very much’ before ‘little’. Still on the studied locations, while more respondents at Palermo and Naples indicated ‘very much’ (but with a wide variability of response), Rome, Milan, and Turin similarly indicated ‘enough’ knowledge about health risks that associate with chemical additives of crustacean products. By response weightings, some respondents indicated ‘no idea’, which suggests such group may be among the less educated of the sampled (usual or unusual) population that consumed crustacean product that lack knowledge about the negative effects associated with the use of chemical additives. If this were to be the case, there is need for improved public awareness about the potential hazards as well as greater caution regarding chemical additives applied to crustacean products. Connecting the respective variables and CV values, higher degree of variability can be seen at those that had ‘no idea’ about reasons that limit their consumption of crustacean product as well as those that indicated ‘not at all’ for when asked whether ‘crustacean with chemicals were unhealthy?’.

### Consumer sensitivity of chemical against non-chemical (MAP) treated crustacean products by price comparisons

Previous sections have dealt with consumer demographic characteristics, consumer types, consumption frequency, and quality ascribed to crustacean products; then reasons for consumption, purchase places and quantity, and perception to chemicals, as well as consumption limitations and perception to chemicals, for the respective usual and non-usual consumers. Again, to reiterate here, the authors deemed to lay foundational knowledge of great essence in order to create a gateway into further research openings, for example, consumer sensitivity of chemical against non-chemical-treated (MAP) crustacean products. The consumer sensitivity by price comparisons of chemical-treated and non-chemical-treated (MAP) crustacean products between usual and non-usual consumers across locations are given in Table 5. For both consumers’ categories, two resembling price scenarios comparing crustacean products treated with chemical additives with those of non-chemical-treated (MAP) using either 15% or 25% price increases are respectively presented. Consistent with the response weightings, it appears fairly obvious that consumers favored the non-chemical products without chemicals and roughly ranged between 83 and 98% regardless of 15% price increase. However, in the situation of price increases of MAP crustacean product by 25%, the response regardless of consumer type, that is, usual and non-usual, would somewhat decrease to range of between 60 and 85%, which would result in some respondents opting out in favor of either chemical-treated crustacean or another seafood product, respectively (Table 5). The determination of price needs special attention because of the high price reactivity of consumers as it can likely bring about a massive substitution effect. Attitude, experience, and knowledge of consumers/individuals capably influence judgment, reasoning, and thinking (22). Besides, if more alternatives of fishery products are available to the consumers, there are more likely to choose other related substitutes (10). As any price increase of non-chemical preserved products may likely lead to a good proportion of usual consumers to potentially increase their budget in the favor of non-chemical, other respondents may equally reduce their purchase frequency/quality but yet, maintain their budget. In this context, the willingness to pay for a given seafood product might associate with consumer preference through cognitive/rationale and symbolic/emotional attributes (10). Thus, if considerable proportion of regular consumers of fishery products were more likely to favor unpackaged seafood products, they may likely consider it less expensive with some additional guarantee for freshness (35). Connecting the respective variables and CV values, the following can be deduced: 1) With regard to the usual consumers, there was high variability when the prices of either €15 or €25 were presented to the consumers and not necessarily at the increase. This may suggest that there could be more unity in response when the price of crustaceans would increase: 2) With regard to the usual consumers as well, when asked what they would do in the situation of increased price of non-chemical (crustacean) product, there was high variability at those that indicated they would ‘reduce purchase quality and maintain budget’; 3) With regard to both price scenes presented to the unusual consumers, the latter showed higher variability consistent with choice of ‘another food product’.

**Table 5:** Consumer sensitivity by cost comparisons between chemical-treated and MAP crustacean products by usual and non-usual (occasional) consumers across locations

Variable	Variable category	Variable sub-category	*n*	Palermo (%)	Naples (%)	Rome (%)	Milan (%)	Turin (%)	Mean (%)	CV	*p*^a^
Usual consumers	Scene 1	Crustacean + chemical additives at €15/kg	24	2.7	2.9	2.0	5.5	6.5	3.92	50.0	>0.05
		Crustacean + MAP at €15/kg×15% extra	632	97.3	97.1	98.0	94.5	93.5	96.08	2.0	
	Scene 2	Crustacean + chemical additives at €25/kg	173	23.5	27.5	29.3	18.8	34.8	26.78	22.5	>0.05
		Crustacean + MAP at €25/kg×25% extra	483	76.5	72.5	70.7	81.2	65.2	73.22	8.2	
	MAP crustacean product with higher	Increase budget for MAP crustacean product	281	43.2	29.0	50.6	48.4	54.3	45.1	21.9	>0.05
	price, what to do?	Reduce purchase frequency and maintain current budget	172	34.3	12.3	22.7	28.1	38.0	27.08	37.4	
		Reduce purchase quality and maintain current budget	184	20.6	54.3	25.3	21.1	15.2	27.3	56.8	
		No idea	17	2.1	4.3	1.3	2.3	3.3	2.66	43.7	
Non-usual consumers	Scene 1	Crustacean + chemical additives at €15/kg	26	2.0	2.9	3.9	4.0	2.7	3.10	27.3	>0.05
		Crustacean + MAP at €15/kg×15% extra	715	96.0	95.2	92.7	87.0	83.2	90.82	6.1	
		Another seafood product?	52	2.0	1.9	3.4	9.0	14.1	6.08	87.9	
	Scene 2	Crustacean + chemical additives at €25/kg	109	5.9	8.7	11.6	18.0	20.1	12.86	47.0	>0.05
		Crustacean + MAP at €25/kg×25% extra	582	79.2	85.6	80.7	65.5	60.4	74.28	14.5	
		Another seafood product?	102	14.9	5.0	6.9	16.5	19.5	12.56	50.1	

*n*=sum of respondents per variable category.

a Outcome of K-W analysis of variance test of variables across locations. CV, coefficient of variation; MAP, modified atmosphere packaging.

### Relationships between variables

For the reason that Kruskal-Wallis (K-W) ANOVA tests revealed no statistical differences comparing locations per variable categories of this study (Tables 1–4), the authors deemed it needful to seek whether these tested variables might exhibit any statistically significant association(s). This involved arranging all locations into one unit per variable to ensure that all locations were considered within each variable to traditionally reduce bias and improve statistical robustness especially in the situation where null hypothesis (H_0_) would be rejected at probability level of *p*<0.05. We found some statistical associations between these tested variables. For instance, the lower the ages of sampled population would show increased cognizance about quality attributes associated with crustacean products (*r*=−0.38, *p*=0.04), which might have likely contributed to increase the reasons that limited them from consuming crustacean products (*r*=−0.462, *p*=0.01). The result tends to corroborate with previous reports that women between 20 and 50 years of age considered fishery products quite expensive overall and this aspect negatively affected its consumption (37). Interestingly, frequent consumers of crustacean products may have fewer reasons that limited their consumption (*r*=−0.337, *p*=0.047). Besides, the consumption frequency increased with decrease in cognizance meted to the quality attributes of crustacean products (*r*=−0.48, *p*=0.001). In addition, the more consumers emphasized on quality attributes of crustacean products, the more it likely limited their consumption (*r*=0.725, *p*<0.001). As anticipated, educational level increased with knowledge about chemical additives posing danger (*r*=0.53, *p*=0.006), worsening taste (*r*=0.79, *p*<0.001), and odor (*r*=0.74, *p*<0.001) of crustacean products. Besides, knowledge required for seafood consumption could be progressively acquired through experience (10). Although knowledge is deemed indispensable, it may not necessarily guarantee behavioral change (22, 38). Likewise, the greater the household composition/number, the less there would likely be emphasis on chemical additives posing danger (*r*=−0.46, *p*=0.02) and worsening odor (*r*=−0.44, *p*=0.03) but would increase the quantity of crustacean products purchased (*r*=0.68, *p*<0.001). As consumers perceived crustacean products more as unhealthy because of presence of chemicals, the less they purchased it (*r*=−0.50, *p*=0.01). If the place of purchase were to be less expensive, the quantity of purchase would likely go up (*r*=−0.58, *p*=0.002), which may directly link to how respondents feel about chemical additives applied to crustacean product posed danger (*r*=0.65, *p*<0.001) and worsened taste (*r*=0.53, *p*=0.007). Although those that viewed crustacean products treated with chemicals were unhealthy believed it posed danger (*r*=0.688, *p*<0.001), the response of it to pose danger was strongly correlated with worsening both taste (*p*<0.001; *r*=0.78) and odor (*p*<0.001; *r*=0.65). In addition, chemical additives that would worsen taste also can directly worsen the odor (*p*<0.001; *r*=0.83).

### General remarks about (specific) theme responses

**Fig. 1:**
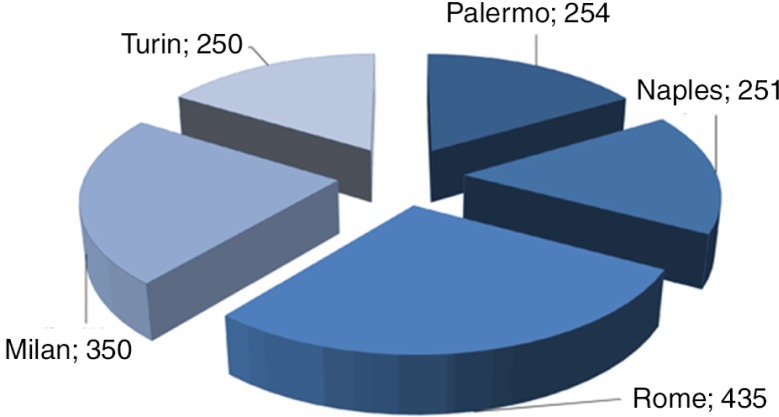
The distribution of respondents by location.

**Fig. 2:**
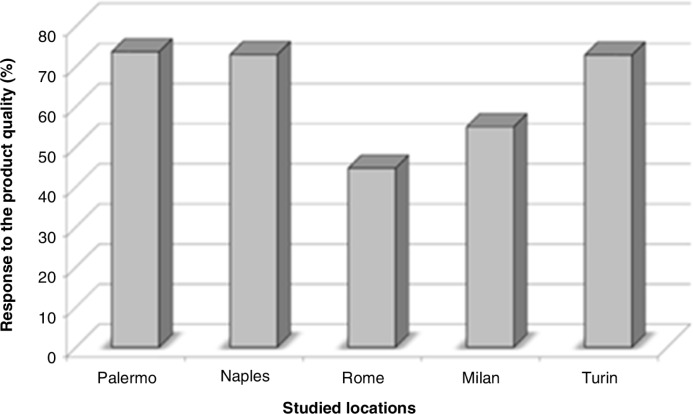
The response rate to ‘quality attributed to or associated with crustacean products by location’.

**Fig. 3:**
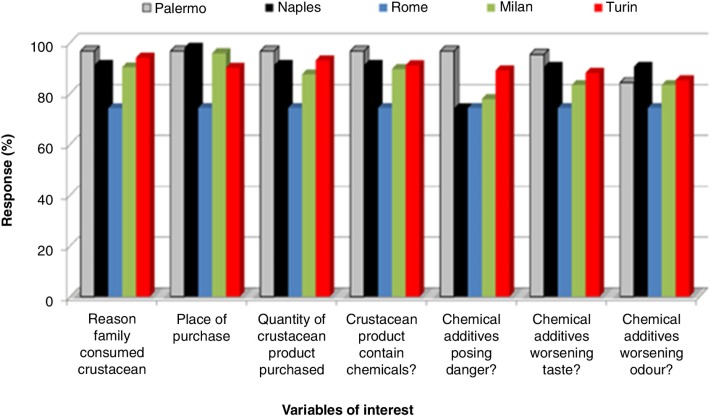
The response rate of usual consumers to various themes by location.

This section deals with how participants of this study responded to some major themes. By total distribution (Fig. 1), Rome appeared with the highest number of participants as would be anticipated considering the location population density, whereas Turin appeared the least. The response to quality attributes of crustacean products by location is given in Fig. 2. Specific to this theme/question, Palermo (73.6%) obtained highest response followed by Naples (72.9%) before Turin (72.8%). The response of usual consumers of crustacean products to other major themes such as reason family consumed crustacean product, place of purchase, quantity of purchase, crustacean product containing chemicals, chemical additive posing danger, worsening odor or taste, by location are given in Fig. 3. We can see that although the responses at Rome interestingly seemed unchanged, it consistently remained the lower across variables of interest of this study. Responses about reason family consumed crustacean product, place of purchase, quantity purchased, crustacean product containing chemicals, and chemical additives posing danger also appeared consistent at Palermo. At Naples, the responses seemed the greater for place of purchase of crustacean product but much less for chemical additives posing danger. While the responses of ‘place of purchase’ showed highest at Milan, the ‘reason family consumed crustacean products’ appeared much less at Turin.

## Research conclusions

The present work was started to establish foundational knowledge with regard to consumers of crustacean products up to consumer sensitivity to price using Italy as a reference case, which can be applied to other parts of the globe. Although there remain questions and challenges yet unanswered, this is the first major step, which eventually will add to the extant body of knowledge regarding consumer sensitivity. This is the first report that attempted a foundational investigation targeting consumer sensitivity of chemical against non-chemical crustacean products using Italy as a case study. Consistent with the response weightings, the conducted investigation found that, as per consumer sensitivity *via* price comparisons, a reasonable proportion of respondents would favor the chemical-free (MAP) crustacean product regardless of 15% price increase. If the price of this chemical-free product were to be further increased by 25%, the proportion of respondents would decrease to between 60 and 85%, which in this case and considering the demand particularly elastic to price changes would result in respondents opting out in favor of either the chemical-treated or another seafood product, respectively. Comparing locations, all studied variables appeared with similar patterns. Nevertheless, the authors believe that differences in participants’ cultures/traditions, educational levels, and standard of living, as well as proximity to coastlines by respective locations might have greatly influenced the nature of responses. In addition, ‘taste’ variable received the highest response followed by ‘family tradition’, thereafter ‘it's healthy’ and less ‘nutritional value’, which were indeed interesting reasons why usual consumers consumed crustacean products. Importantly, further studies are still required to better understand the specific price elasticity of crustacean demand, which is crucial to give more detailed conceptual and practical guidance to the industry. A number of tested variables showed both positive and negative statistical correlations regardless of locations. For example, it was found that younger age of the sampled population would increase cognizance about quality attributes associated with crustacean products to likely account for increased reasons to limit their consuming crustacean products. As anticipated, the educational level of respondents increases with knowledge about chemical additives. Moreover, respondents agreed that chemical additives that posed danger to crustacean products would directly worsen its taste and odor.

## Research limitations and future outlooks

Whether consumers decide to either purchase or not purchase crustacean products appears not tackled in this study. This could be a limitation and future work should be directed to delineate this. The strong descriptive approach used to perform this study to compare consumer types by different regions of Italy may make it look rather localized. This thus necessitates additional studies in this direction at other parts of globe. The data generated from such studies would help supplement the current information. In addition, more research should be directed to other sensitive areas that affect usual and unusual consumers of crustacean products, for example, health, hygiene, product distribution, and accessibility of processed product comparing chemical-treated with non-chemical preservation methods, such as MAP.
